# Optic nerve head sarcoidosis mimicking an intraocular tumour, and occurring as the first manifestation of neuro-ocular sarcoidosis

**DOI:** 10.1016/j.ajoc.2020.100988

**Published:** 2020-10-29

**Authors:** Yamini Krishna, Louise Christou, Jakub Khzouz, Rumana Hussain, Heinrich Heimann, Sarah E. Coupland

**Affiliations:** a Liverpool Clinical Laboratories, Liverpool University Hospitals NHS Foundation Trust, Liverpool, UK; b Liverpool Ocular Oncology Research Centre, Department of Molecular and Clinical Cancer Medicine, University of Liverpool, Liverpool, UK; c St Pauls Eye Clinic, Liverpool University Hospitals NHS Foundation Trust, Liverpool, UK; d Liverpool Ocular Oncology Centre, Liverpool University Hospitals NHS Foundation Trust, Liverpool, UK

**Keywords:** Neuro-ocular sarcoidosis, Optic nerve sarcoidosis, Ocular sarcoidosis, Mediastinal sarcoidosis, Hodgkin lymphoma, Sarcoid-like reaction

## Abstract

**Purpose:**

Sarcoidosis is a chronic idiopathic granulomatous inflammatory disease that can affect many major organ systems, primarily the lungs, and hence has remarkable clinical heterogeneity. At least 50% of patients with systemic sarcoidosis develop inflammatory eye disease, and in approximately 21% of cases, it may be the first clinical manifestation. Neuro-ocular involvement occurs in <3% of all sarcoidosis cases, and rarely involves the optic nerve. We describe an unusual case of an intraocular sarcoidosis presenting as an unclear optic nerve mass.

**Observations:**

A 61-year-old male presented with painful gradual visual loss in the right eye. Previous history included Stage II Hodgkin lymphoma (HL) and concurrent mediastinal sarcoidosis, both in remission 5 years later. On examination, the right eye (RE) vision had no light perception, neovascular glaucoma, attenuated retinal vessels and a non-pigmented optic disc mass. The left eye was normal. The RE showed no response to oral steroids, was painful due to neovascular glaucoma and the concerns of recurrent HL with intraocular manifestations lead to RE enucleation. Macroscopic examination revealed a whitish mass at the optic disc, which histomorphologically showed a non-necrotising granulomatous inflammation consuming the optic nerve head and extending into the optic nerve resection margin. Special stains for microorganisms were negative. The uveal tract was free of inflammation. The morphological features were consistent with optic nerve sarcoidosis. A diagnosis of neuro-ocular sarcoidosis was made, and the patient was commenced on infliximab.

****Co**nclusion:**

Neuro-ocular sarcoidosis is known as the ‘great imitator’ because it can cause a variety of non-specific clinical signs and symptoms, mimicking many other conditions, including lymphomas. Intraocular sarcoidosis is not unusual and typically affects the uvea. Isolated optic nerve sarcoidosis is rare. The challenging aspect of intraocular sarcoidosis is the requirement of prompt treatment to reverse any eye damage and prevent permanent visual loss. Here, optic nerve sarcoidosis was very advanced, and was associated with intracerebral manifestations.

**Importance:**

Neuro-ocular sarcoidosis is a difficult condition to diagnose and treat. Our case was complicated by the previous history of HL and concurrent mediastinal sarcoidosis which were in remission. In patients with a history of sarcoidosis with new loss of vision and neurological weaknesses oculocerebral involvement must be included in the differential diagnosis even in the absence of typical manifestations of ocular sarcoidosis as in uveal tract involvement.

## Highlights

• Optic nerve head (ONH) sarcoidosis without uveal tract involvement is rare.
• ONH sarcoidosis can be the first manifestation of systemic sarcoidosis.
• ONH sarcoidosis may be the first manifestation of neuro-ocular sarcoidosis.
• ONH masses present diagnostic challenges due to the impact of tissue biopsy.
• ‘Sarcoid-like’ reaction or sarcoid-lymphoma syndrome may occur with concurrent lymphoma.

## 1 Introduction

Sarcoidosis is a chronic idiopathic granulomatous inflammatory disease that masquerades many other conditions. Sarcoidosis can affect many major organs although it predominantly affects the pulmonary system.^1^ Intraocular sarcoidosis has a reported incidence varying considerably between 13 and 79%,^2^, ^3^, ^4^, ^5^ and indeed it is the first clinical manifestation of the disease in 21% of cases.^1^ Uveal tract involvement is the most common ocular finding. Neurosarcoidosis occurs in 5–10% of systemic sarcoidosis cases.^6^ The combination of both neurological and ocular involvement occurs in 2%–3% of all sarcoidosis patients, with optic nerve involvement rarely including optic nerve head (ONH) granulomata.^6^

Herein we describe an unusual case of an isolated ONH tumour presenting as the first manifestation of recurrent systemic sarcoidosis and as the initial manifestation of neuro-ocular sarcoidosis in a male patient with a background history of Hodgkin lymphoma (HL).

### 1.1 Case report

A 61-year old man was referred to the Liverpool Ocular Oncology Centre (LOOC) in 2018 with a right optic nerve head (ONH) mass. He had a one year history of monocular visual loss and increasing pain in the right eye that had not responded to a four-week course of oral corticosteroids. His previous ophthalmic and general medical history included: Stage II HL involving the left cervical nodes diagnosed in 2013, treated with four cycles of chemotherapy (Adriamycin, Vinblastine, Dacarbazine; AVD) and field radiotherapy; mediastinal sarcoidosis suspected on an interim positron emission tomography scan during chemotherapy, and confirmed by mediastinal lymph node biopsy in 2014; as well as diabetes mellitus and systemic hypertension.

**Fig. 1:**
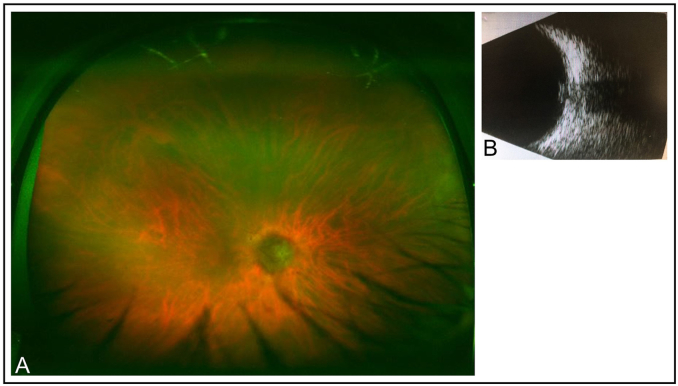
A)Colour fundus photograph of the right eye showing a non-pigmented optic disc lesion and attenuated retinal vessels. B) B-scan image of the tumour-like optic nerve head lesion. (For interpretation of the references to colour in this figure legend, the reader is referred to the Web version of this article.)

The patient's medication at the time of LOOC consultation included latanoprost, iopidine, atropine drops to the right eye; amlodipine; ramipril and aspirin. On ophthalmological examination, vision in the left eye (LE) was 6/6 aided; anterior segment examination was normal with an intraocular pressure (IOP) of 19 mmHg. There was no light perception (NLP) in the right eye (RE), mild conjunctival injection, a clear cornea with a deep and ‘quiet’ anterior chamber, rubeosis iridis and anterior synechiae. His right IOP was 41 mmHg. The right anterior vitreous was unremarkable. Fundoscopy of the RE revealed attenuated retinal vessels and a non-pigmented mass at the optic disc (Fig. 1a). B-scan of the optic nerve demonstrated a tumour-like lesion of the ONH extending beyond the lamina cribrosa (Fig. 1b). The LE fundus was normal.

The differential diagnoses included: circumscribed intraocular lymphoma, amelanotic melanoma or intraocular sarcoidosis. Given the finding of a steroid unresponsive blind and painful RE, the patient agreed to RE enucleation with implantation of an acrylic prosthesis. The specimen was sent in buffered formalin for histopathological examination.

Macroscopic examination revealed an intact, right globe with dimensions of 24.5mm (axial) x 23.3mm (horizontal) x 23.5mm vertical. The cornea was clear and measured 12mm horizontally and 11.5mm vertically. The optic nerve was 4mm in length. There was no shadow noted on *trans*-illumination of the eye prior to opening. On dissection of the eye, there was a white-yellow mass measuring 3.3 × 4mm at the ONH.

**Fig. 2:**
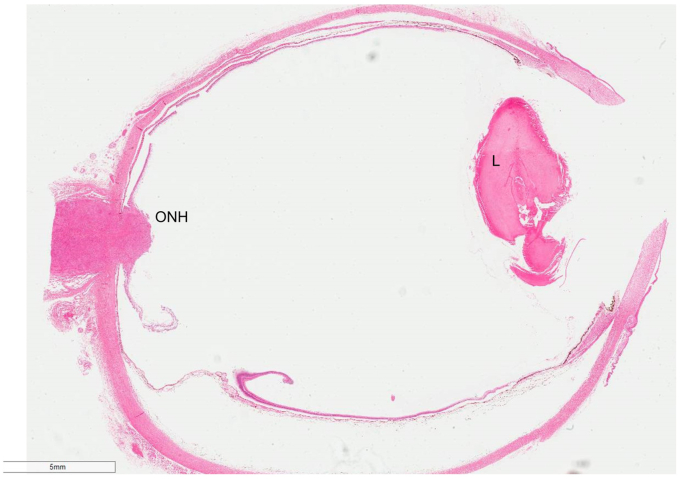
Haematoxylin & Eosin (H&E) photomicrograph of the eye showing an enlarged optic nerve head (ONH). Lens (L).

**Fig. 3:**
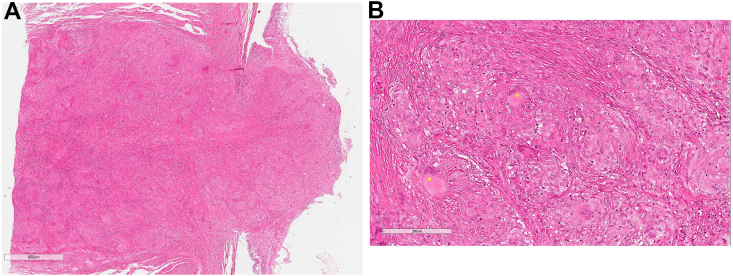
A) ‘Mushroom-like’ lesion at the ONH (H&E) with B) numerous granulomata containing multinucleated Langhans-type and foreign-body type giant cells (*). Infiltrates extend to the optic nerve resection margin.

**Fig. 4:**
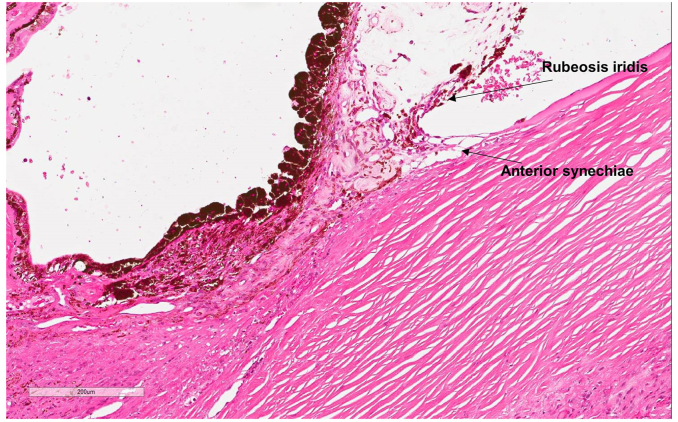
The anterior segment with iris neovascularisation and very fine anterior synechiae.

Histomorphological findings of the RE included ONH and nerve replacement by an extensive non-caseating granulomatous infiltrate (Fig. 2). The latter was mainly composed of epithelioid histiocytes with abundant eosinophilic cytoplasm and scattered plasma cells, eosinophils and small reactive lymphocytes. Many of the epithelioid histiocytes were arranged in confluent granulomas composed of multinucleated cells forming Langhans’-type giant cells (Fig. 3a and b). The granulomatous infiltrate extended into the optic nerve excision margin (Fig. 3a). It was limited to the optic disc and did not involve either the adjacent retina, sclera or choroid. In the anterior segment of the eye, the iris showed neovascularisation with very fine synechiae between the iris leaves and the posterior cornea (Fig. 4). The lens showed age-related cataractous changes. Despite multiple levels being undertaken, granulomata were not seen in the entire uveal tract.

**Fig. 5:**
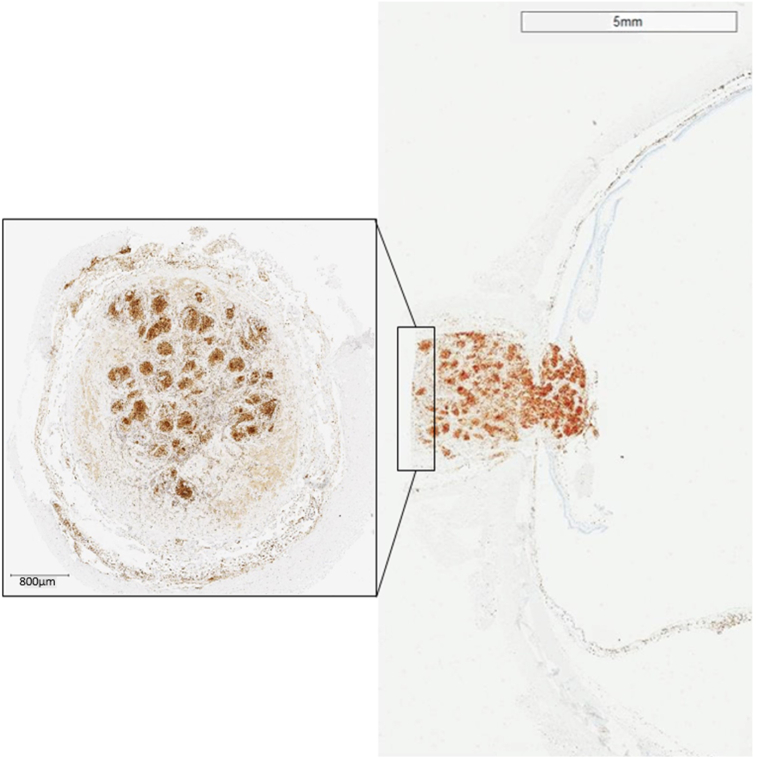
CD68PG immunostain highlighting numerous histiocytes within the almost confluent granulomata in the optic nerve, and extending into the surgical resection margin (square).

Special stains of the optic nerve lesion for acid fast bacilli (Ziehl-Neelsen) and fungi (Periodic Acid Schiff and Grocott-Gomori's methenamine silver) were negative. The CD68 immunostain highlighted the numerous histiocytes and giant cells (Fig. 5). The morphological appearances were consistent with the diagnosis of optic nerve sarcoidosis. There was no evidence of malignancy; in particular, there was no evidence of an intraocular manifestation of the patient's HL.

Simultaneous to the histomorphological investigation of the RE, a recurrence of mediastinal lymphadenopathy was observed on the patient's computed tomography of the chest. The differential diagnoses were recurrent HL or mediastinal sarcoidosis. The multi-disciplinary team concluded that the radiological features of widespread lymphadenopathy involving hilar and mediastinal regions plus axillary-, iliac- and inguinal nodes together with sub-central bilateral lung nodules were all in keeping with sarcoidosis. The ultimate histological diagnosis of the ONH ‘tumour’ in the RE supported the clinical diagnosis of recurrent systemic sarcoidosis.

The patient was discharged to a Hospital Trust closer to his home residence, and joint management of the systemic sarcoidosis was continued by Haematological-, Respiratory Medicine and Neurological teams at this site. In 2019, unfortunately, the patient developed increasing right-sided lower limb weakness, imbalance and worsening tremulousness in the arms and head. Oral prednisolone 45mg was started for six weeks with some improvement in symptoms, but a subsequent referral to a tertiary neuro-sarcoidosis clinic for commencement of infliximab was organised.

## 2 Discussion

Here we present a very unusual case of histomorphologically-confirmed ONH sarcoidosis in a patient with a background history of concurrent HL and mediastinal sarcoidosis, considered to be in remission. Neuro-ophthalmic sarcoidosis presenting initially as isolate optic nerve granulomata, without any involvement of the uveal tract, is rare. In addition, our case demonstrates the diagnostic uncertainties faced in determining recurrent sarcoidosis versus a recurrent lymphoma. The case exemplifies coexistent pathologies, termed sarcoid-like reactions or ‘sarcoid-lymphoma syndrome’, which are not well understood.^7^

Sarcoidosis is a chronic idiopathic granulomatous inflammatory disease that can affect any major organ system, primarily the lungs, and hence has remarkable heterogeneity in clinical presentation, findings and natural history.^8^ The incidence of ocular involvement in systemic sarcoidosis is variable according to different studies, ranging from 13% in a Turkish study,^2^ 21% in Korean study^4^ to 53% in a British study,^9^^,^^10^ through to 79% in Japanese study.^5^

In approximately 21% of patients, ocular sarcoidosis may be the first clinical manifestation of the systemic disease.^1^ Lower incidences have been reported in two large multi-centred studies in America (ACCESS, 12%) and Europe (GenPhenReSa, 8%).^11^^,^^12^ Ocular manifestations of sarcoidosis are non-specific with the most frequent clinical findings of uveitis, dry eye and conjunctival nodules.^13^ The uveal tract is most commonly involved, although any segment of the eye and/or orbital structures can be affected. Uveitis can be anterior, intermediate, posterior or pan-uveitis. Clinical findings include ‘mutton-fat’ keratic precipitates, iris or trabecular meshwork nodules (granulomas), vitritis with snowball formation, and posteriorly; periphlebitis, chorioretinitis and choroidal granulomas. Other findings of ophthalmic sarcoidosis also include lacrimal gland swelling and conjunctival granulomas. Pasadhika and Rosenbaum^13^ summarise the ocular manifestations of sarcoidosis, highlighting that any part of the eye or adnexae may be involved (Table 1).

**Table 1:** Clinical manifestations of ocular sarcoidosis summarised by Pasadhika and Rosenbaum.^13^

Ocular structures	Ophthalmic manifestations
Eyelids	Eyelid granuloma, madarosis, poliosis, entropion, trichiasis, lagoghthalmos (if associated with facial palsy)
Conjunctiva	Conjunctival nodules or granuloma, conjunctivitis, symblepharon, conjunctival cicatrization
Episclera/sclera	Episcleritis, scleritis
Cornea	Peripheral ulcerative keratitis, interstitial keratitis, exposure keratopathy, band keratopathy
Trabecular meshwork and anterior chamber angle	Trabecular granuloma, peripheral anterior synechiae, ocular hypertension, glaucoma
Iris	Anterior uveitis (iritis), iris nodules/granuloma, posterior synechiae, pupillary abnormalities
Lens	Cataract
Pars plana/vitreous	Intermediate uveitis
Retina	Retinitis, retinal vasculitis, macular edema
Choroid	Choroiditis, granuloma
Lacrimal gland	Granuloma, dacryoadenitis, keratoconjunctivitis sicca (dry eye)
Nasolacrimal drainage system	Nasolacrimal duct obstruction
Extraocular muscles and other orbital tissues	Granuloma, strabismus, proptosis, optic nerve compression
Intracranial lesions involving visual pathway	Decreased vision, visual field defects, abnormal pupillary response, abnormal eye movement

Optic disc granulomata are very rare manifestations of sarcoidosis and occurs in less than 3% of patients with sarcoidosis.^1^ In a literature review by Hickman et al., 34 cases of ONH granuloma were previously reported.^14^ Only two of the 34 cases report histologically-confirmed diagnosis as enucleation was performed and one post-mortem examination.^15^^,^^16^ Each case demonstrated non-caseating granuloma, however, involvement was not confined to the ONH. Infiltration into the vitreous, sclera and/or retina was also seen.

Subsequent to Hickman's review of the literature, there are eight further cases of ONH granuloma secondary to sarcoidosis reported in the English literature.^17^, ^18^, ^19^, ^20^, ^21^, ^22^, ^23^ One case reports an optic nerve biopsy; however, further clinical details are not described.^17^ Another case underwent intracranial optic nerve ‘shave’ biopsy.^22^ Two cases had no biopsy but since there was overwhelming clinical evidence of systemic sarcoidosis, it was assumed that the ocular lesions were also caused by the disease.^18^ Four had extraocular biopsies only (three mediastinal and one brain parenchymal).^19^, ^20^, ^21^^,^^23^

In our case, enucleation was performed due to a blind painful eye unresponsive to steroids. There was extensive infiltration of the optic nerve and involving surgical resection margin by non-caseating epithelioid granulomata, whereas the rest of the eye was ‘quiet’. In contrast to the histology reported in previous cases,^15^^,^^16^ the granulomatous infiltrate was confined to the optic nerve without any evidence of involvement of the uveal tract. Review of the previous cases indicates that optic nerve granuloma is an infrequent finding, and biopsy is obviously not often feasible. Awareness of cases such as ours, with granuloma confined to the optic nerve, as well as the absence of optic nerve biopsies in many other instances, highlights the diagnostic uncertainty faced in the absence of histological confirmation. Furthermore, our case provides a reminder of the differential diagnosis of neuro-sarcoidosis in optic nerve lesions. This is of particular importance to future practise as intraocular sarcoidosis does not necessarily always have to involve the uveal tract, as written almost as dogma in many Ophthalmology textbooks. This case also justifies consideration of optic nerve biopsy in the context of severe sight-threatening optic-nerve lesions.

Neurosarcoidosis occurs in 5–10% of systemic sarcoidosis cases,^6^ and presents with variable symptoms: half of the patients present with a simple cranial neuropathy affecting one or more of the cranial nerves (e.g. CNIII, VI, VII and VIII). Of the remaining half of patients, two thirds have leptomeningitis, one quarter pachymeningitis, and the remainder the vasculitic form. These cases are much more serious and require urgent evaluation and treatment.^10^^,^^11^ Neuro-ophthalmic sarcoidosis is a specific entity in which there is inflammatory involvement of the optic nerve up to the chiasm (anterior visual pathway) and/or involvement of oculomotor, trochlear and abducens cranial nerves. It typically presents as visual loss (central or peripheral) or diplopia. The frequency of neuro-ophthalmic sarcoidosis has not been extensively reported; however, one study suggests that it affects 2–3% of all sarcoidosis patients.^6^^,^^24^^,^^25^

Since neuro-ophthalmic sarcoidosis is rare; there are no agreed treatment guidelines. Systemic corticosteroid is the first-line therapy, and results from different studies showing variable patient response.^10^^,^^25^ Other steroid-sparing medications should be considered where long-term therapy is probable to avoid side effects from prolonged steroid use or in refractory cases of neurosarcoidosis after initial treatment with systemic corticosteroids. Alternatives to corticosteroids include cytotoxic medications such as methotrexate, azathioprine, mycophenolate, intermittent intravenous cyclophosphamide, and some Tumour Necrosis Factor inhibitors (e.g. Infliximab and Adalimumab).^6^^,^^12^^,^^24^ Our patient was treated with a course of oral prednisolone without functional improvement during the early stage of monocular vision loss and prior to confirmation of neuro-ophthalmic sarcoidosis. Subsequent to the RE enucleation, oral corticosteroids were trialled for systemic neuro-sarcoidosis with good effect leading to consideration for treatment with infliximab.

Finally, our case was of further interest due to the complexity in reaching a diagnosis due to the patient's background of concurrent cervical HL and mediastinal sarcoidosis. Confusing overlap exists between the clinical manifestations of HL and sarcoidosis leading to challenges in distinguishing the two conditions. Terms to describe the occurrence of HL and sarcoid concurrently or one after the other have been proposed. ‘Sarcoid-like’ reaction refers to the development of non-caseating granulomas in patients with a known malignancy (e.g. various solid cancer and haematological malignancies).^26^ A ‘sarcoid-like’ reaction has been observed in organs distant from the primary malignancy as well as in lymph nodes that drain the neoplastic tissue.^27^ In contrast, ‘Sarcoid-lymphoma’ syndrome refers to the development of lymphoma in patients with sarcoidosis. The co-existence of HL and sarcoid is also documented in both ‘sarcoid-like’ reactions and ‘sarcoid-lymphoma syndrome,‘^7^^,^^28^ unfortunately with confusing interchangeability in the literature. Each condition is not fully understood, although may be associated with conditions of chronic inflammation and dysfunction in immunoregulatory pathways.^26^ A literature review reveals limited discussion of ‘sarcoid-like’ reaction or ‘sarcoid-lymphoma’ syndrome with ocular involvement.^29^^,^^30^ Our case demonstrates some more commonly identified characteristics of these paraneoplastic phenomena such as the association with lymphoma rather than solid organ malignancies and concurrence of the inflammatory and malignant processes. However, our case is a novel example of a sarcoid-like reaction due to the involvement of the optic nerve compared to other cases that describe inflammatory involvement of the vitreous, retina and choroid. Awareness of the poorly understood paraneoplastic phenomena is important to avoid overlooking an inflammatory condition in the presence of biopsy-confirmed malignancy and vice versa.

## 3 Conclusion

Isolated optic nerve sarcoidosis is a rare finding, which may mimic other intra-ocular tumours and result in total loss of vision, and led to enucleation in our case.

Biopsy is the most accurate way to confirm the diagnosis; however, biopsy of intraocular tissue or neural tissue is usually not performed due to the invasiveness and impact of optic nerve biopsy. In contrast to the few cases reported, we present findings of sarcoid-related granulomatous infiltrate confined to the ONH and absent in other ocular tissues. Our case also demonstrates the poorly understood phenomenon of sarcoid-lymphoma syndrome.

## Patient consent

Written consent for the anonymous use of clinical information and biosamples was obtained from the patient. This documentation can be provided on request.

## Funding

No funding or grant support

## Authorship contributions

All authors attest that they meet the current ICMJE criteria for Authorship.

## Authship statements

Yamini Krishna: Investigation, Writing, review & editing, Visualization. Louise Christou: Investigation, Writing, review & editing, Visualization. Jakub Khzouz: Visualization, Writing. Rumana Hussain: Patient care; Conceptualization, Investigation. Heinrich Heimann: Patient care; Conceptualization, Supervision. Sarah E. Coupland: Conceptualization; Writing, review & editing; Supervision.

## Declaration of competing interest

The following authors have no financial disclosures: YK, LC, JK, RH, HH, SEC.
